## [Editor Report]

This fundamental work integrates transcriptome and proteome analysis across human neurogenesis, uncovering posttranscriptional regulatory mechanisms for a specific gene module enriched in ribosomal genes. The evidence supporting the conclusions is compelling, exploiting a range of targeted human pluripotent stem cell lines for brain organoid generation. The work will be of broad interest to developmental and neurobiologist.

---

## [Decision Letter]

**Decision letter after peer review:**

Thank you for submitting your article "Integrated transcriptome and proteome analysis in human brain organoids reveals posttranscriptional regulation of ribosomal genes" for consideration by *eLife*. Your article has been reviewed by 3 peer reviewers, including Anita Bhattacharyya as the Reviewing Editor and Reviewer #3, and the evaluation has been overseen by a Reviewing Editor and Marianne Bronner as the Senior Editor.

Essential revisions:

1) Replication of key findings/validation in at least one independent cell line. The data is from organoids from one human stem cell line, the female H9 human embryonic stem cell line, and so it is critical to validate the results on 1-2 additional stem cell lines, to rule out the possibility that these results are unique to this one cell line/individual.

2) Verification of stress response in primary tissue. The authors identified a stress-related role for processes in corticogenesis, although, without comparison to human tissue, it's possible that some of the results are due to the artificial nature of the organoids as at least some human brain organoid protocols have been reported to lead to ectopic activation of cellular stress pathways (Bhaduri et al., Nature 2019). This aspect of the study should be confirmed in primary tissue (mouse or human).

3) Modify claims or add additional data to address Reviewers' comments. There are several instances in which the authors make statements that are not supported by the data or neglect previously published data, as detailed in the reviews below.

a) Additional discussion or experiments should convey the impact of the results on progenitor subtypes and highlight the fact that oRGs are a population that is uniquely subject to mTOR activation.

b) Many RBPs have an important role in the regulation of mRNAs during brain development and neuronal differentiation (and are implicated in developmental disorders), including several of the proteins/families listed in Figure 3C. Please provide some examples and references to identify specific examples of proteins in the list that may play a role.

c) The results themselves do not suggest that the length of neuron-enriched genes contributes to their complex regulatory mechanisms, but add to the existing literature that should be cited

https://www.nature.com/articles/nature12504

https://academic.oup.com/bioinformatics/article/34/13/i422/5045809

https://pubmed.ncbi.nlm.nih.gov/25232122/

https://www.nature.com/articles/nature14319

https://www.ncbi.nlm.nih.gov/pmc/articles/PMC3638137/

d) There are some limitations of the specific reporter and knockout hPSC lines that could be discussed more, as well as providing additional quality control measures including whether the lines remain karyotypically normal throughout the targeting procedure, whether gene expression faithfully recapitulates the activity of the promoters controlling their expression. Specifically, it appears that a significant GFP signal is detected within the neuronal layer (Figure 1B) and that there is a much larger double reporter-positive population than expected (Figure S2A).

*Reviewer #1 (Recommendations for the authors):*

This was a nice paper, and generally quite strong. A few questions/suggestions:

1. Profiling the "in-between" population would be a very interesting and important characterization with regards to the model of mTOR regulation and the progenitor/neuron transition.

2. The normalization is important, but did it impact the results? Can you validate protein levels in the cases where RNA and protein are dysregulated (ie RNA higher but protein lower and vis versa), specifically by looking more expansively at protein levels?

3. Subtypes are critical to cortical development. This approach clearly highlights progenitor/neuronal differences but could the observations be extended to one or two subtypes? Specifically, oRGs come to mind as a population that is uniquely subject to mTOR activation.

4. The TSC experiments are nice, but also are specific to that pathway and manner of regulation of mTOR signaling. It is possible that mTOR broadly has more complex or nuanced impacts upon 5'-TOP regulation. Thus, I would suggest pharmacologically overactivating and repressing it to explore similar impacts, or limiting the degree to which mTOR is described to overall control the regulation of these transcripts.

*Reviewer #3 (Recommendations for the authors):*

1. The authors identified a stress-related role for processes in corticogenesis, although, without comparison to human tissue, it's possible that some of the results are due to the artificial nature of the organoids as they have been reported to have elevated stress (Bhaduri et al.,). The authors should tone down the statement "Our observation of SG-like RNA-granules in early ventricular radial glia further supports the occurrence of stress-associated processes during corticogenesis," in the discussion as the organoid system does not completely mimic corticogenesis.

2. The data is from organoids from one human stem cell line, the female H9 human embryonic stem cell line and so it is critical to validate the results on 1-2 additional stem cell lines, to rule out the possibility that these results are unique to this one cell line/individual.

3. There are several instances in which the authors make statements that are not supported by the data or neglect previously published data.

a) Title:

a. The use of the word "reveals" in the title implies that post-transcriptional regulation of ribosomal genes has not been seen before, which isn't true, since it is established in other cell types. I think instead the title should reflect that the authors have shown posttranscriptional regulation of ribosomal genes in human neural tissues

i. https://academic.oup.com/nar/article/25/5/995/2360248

ii. https://pubmed.ncbi.nlm.nih.gov/11029573/

iii. https://pubmed.ncbi.nlm.nih.gov/18498749/

b. Since the only validation and focus has been on posttranscriptional regulation of ribosomal genes via 5'TOP, the title also seems too broad unless you add validation demonstrating additional mechanisms of transcriptional regulation of these genes

b) Page 11, Lines 5-8, and Page 11 Lines 13-15

"Effective" is not the right word to use here. How can a translation be less effective or more effective? There are more than just the two stated reasons why there could be differences in mRNA transcript level but not protein level in these two modules, some of which are discussed on the next page (page 12, lines 6-10).

c) Page 12, Lines 22-24: "the RBP motifs we identified and their associated proteins might play a prominent role in cell class-specific expression to promote progenitor or neuron-specific gene regulation " Many RBPs have an important role in the regulation of mRNAs during brain development and neuronal differentiation (and are implicated in developmental disorders), including several of the proteins/families listed in Figure 3C. It might be more impactful to specifically provide some examples and references instead of generically stating that the proteins in the list "might" play a role. These references would also strengthen your claims and provide additional evidence that your data and model system are consistent with previously published work in the field.

d) Given that alternative splicing is mentioned in the introduction (page 5, line 9) as something that has a role in determining progenitor fate and neuronal migration in mouse and given that the RBPs in Figure 3C contains several splicing factors/regulators (SRSF family, KHDRBS3, CPEB3, PTBP1, etc.), it might be worth discussing this more or even adding splicing analysis into your paper. It could provide additional clarity about the mRNA and protein levels in the applicable modules.

e) Page 13, Line 1-3

Omit "might" from this sentence because, in the next sentence, you give a very definitive example of a fate regulator that is regulated by miRNAs. You could alternatively say that these data are consistent with miRNA regulation of their expression, for example.

f) Page 13, Line 20-22

These observations themselves do not suggest that the length of neuron-enriched genes contributes to their complex regulatory mechanisms. These observations add to the existing literature that previously suggested this:

https://www.nature.com/articles/nature12504

https://academic.oup.com/bioinformatics/article/34/13/i422/5045809

https://pubmed.ncbi.nlm.nih.gov/25232122/

https://www.nature.com/articles/nature14319

https://www.ncbi.nlm.nih.gov/pmc/articles/PMC3638137/

g) The conclusions about the results of Figure 3 (and its supplements) could likely be strengthened by performing similar analyses on the datasets used for Figure 4 Supplemental Figure 1 (Blair et al. 2017; Eze et al. 2021)

---

## [Author Response]

Essential revisions:1) Replication of key findings/validation in at least one independent cell line. The data is from organoids from one human stem cell line, the female H9 human embryonic stem cell line, and so it is critical to validate the results on 1-2 additional stem cell lines, to rule out the possibility that these results are unique to this one cell line/individual.

We thank the reviewers for raising this important point. We would like to point out that in the original manuscript we already used an additional hiPSC line – Rozh-5 to validate the key findings. In the mentioned figures, we observe the replication of the findings in this additional hPSC background for the pS6 distribution in the tissue (Figure 5A and Figure 5—figure supplement 1F,G), the 5’TOP reporter assay (Figure 4D,E and Figure 4—figure supplement 3) and the impact of TSC2 KO on RPL5 and RPL11 protein distribution (Figure 5G and Figure 5—figure supplement 3). In the revised version we have also validated occurrence of G3BP1 positive stress granule-like structures in the ventricular zone of Rozh5 organoids (Figure 4—figure supplement 4D).

Furthermore, the polysome profiling experiment in the original manuscript in Figure 5 was done in the Rozh-5 background. This was specifically done to address the point of genetic background. The results of the polysome profiling validated our hypothesis on mTOR-mediated regulation. The effect on neurogenesis identified through these experiments was later validated in the H9 dual reporter background (Figure 5—figure supplement 4D,E).

Overall, these results help to reduce the possibility that our observations are unique to one cell line/individual. We apologize that this was not clear in the first version of the manuscript, and we now mention it explicitly in the revised version.

2) Verification of stress response in primary tissue. The authors identified a stress-related role for processes in corticogenesis, although, without comparison to human tissue, it's possible that some of the results are due to the artificial nature of the organoids as at least some human brain organoid protocols have been reported to lead to ectopic activation of cellular stress pathways (Bhaduri et al., Nature 2019). This aspect of the study should be confirmed in primary tissue (mouse or human).

We thank the reviewers for their comment. We would like to raise attention to the following points:

Indeed, the occurrence of stress in the brain organoids has been an important field of investigation. However, we would like to point out that recent publications from the group of Paola Arlotta (Uzquiano et al., Cell 2022; DOI: 10.1016/j.cell.2022.09.010 ) and our own group (Vertesy and Eichmüller, EMBO journal 2022; DOI: 10.15252/embj.2022111118 ) have shown that the ‘stress levels’ are not as broadly distributed as initially proposed by Bhaduri et al. Nature 2019; DOI: 10.1038/s41586-020-1962-0. These studies show that only a minority of cells are present in the so called ‘stressed’ condition, which are mainly located towards the center of the organoid and predominant at 2 month and older stages of organoid growth. Keeping these points in mind, we think our microscopic observations of G3BP1-positive granular structures at day 40 do not focus on particularly stressed cells.Our analyses indicate that the same molecular regulation involved in 5’TOP translational inhibition in acute stress seems to play a role here in early progenitors. However, we do not propose that early progenitors are in a stressed state. In line with this hypothesis, we do not expect and do not observe occurrence of stress granules and other stress feature in every progenitor. While we observe G3BP1 positive stress granule-like structures in the organoids, they are overall rare as seen from the quantification. As a positive control for stress condition, we used sodium arsenite (NaAs) treatment which indeed resulted in very high number of G3BP1 positive granular structures observed almost in every cell. Additionally, in the revised manuscript we analyzed the distribution of phospho-EIF2a, a typical marker of stress response (As reviewed by Pakos-Zebrucka et al., EMBO report 2016 DOI: 10.15252/embr.201642195).

We found that while the tissue in NaAs-treated organoids shows very high levels of phosphoEIF2a, the control organoid tissue shows very low levels of phospho-EIF2a (Figure 4—figure supplement 4E,F). These observations further suggest that the organoid tissue is not under a general acute stress.

1. Another study by Esk et al., Science 2020 DOI: 10.1126/science.abb5390 analyzed ER morphology in the ventricular zone of telencephalic brain organoids grown using the same protocol. In this study the authors found that the control organoids show normal ER morphology, unlike organoids showing unfolded protein response and ER stress caused by loss of IER3IP1 protein. These results further provide another positive control for a stress condition and highlight absence of a general acute stress in brain organoids.

Bhaduri et al. Nature 2019 and the other studies mentioned in the first point conclude stress state based solely on transcriptomic signature and do not include any protein level investigation or cell biological assays to validate if the cells indeed show other signs of stress. Firstly, as pointed out by the reviewers, the general anecdotal evidence in the field and our own study highlights the importance of considering both RNA and protein information. Furthermore, research from the stress biology field indicates that there are important cellular changes independent of transcriptional changes that are exhibited by a stressed cell (protein aggregation, stress granule formation, phosphorylation of EIF2a, ER inflation), which are not directly observed in control organoids. Thus, the conclusion about stress in organoids proposed by Bhaduri et al. Nature 2019 should be considered with caution.

We do not have access to human fetal material where the associated ethical permissions would allow us to perform the suggested experiments at a suitable stage of fetal development. Instead, as suggested by the reviewers we tested occurrence of G3BP1 positive structures in the E12.5 developing mouse brain (Author response image 1). While we could observe a few G3BP1 puncta, these structures were not observed commonly. Hence, it is difficult to state if the same mechanism is at play in the developing mouse brain. There could be species differences due to different subtypes of progenitors and the timescale differences of cell cycle and development. Nevertheless, upon analysing a sc-RNAseq dataset for the developing mouse brain (Telley et al., Science 2019; DOI: 10.1126/science.aav2522 ), we could verify that the RNA difference between progenitors and neurons for 5’TOP genes can also be seen in the developing mouse brain (Author response image 1). This is in line with the analysis of the human fetal scRNA seq data (Figure 4—figure supplement 1F. Eze et al., Nat Neuroscience 2021; DOI: 10.1038/s41593-02000794-1).

Nevertheless, to address the raised concern, in addition to the newly added data mentioned above, we have made several text changes (Page 15, line 14-16; Page 22 line 3-6) to align our conclusions with the observations in the manuscript, including the title of the manuscript. We have explicitly mentioned this caveat and the need to replicate these findings in primary human tissue in a limitations paragraph at the end of the manuscript (Page 25, line 15 to Page 26, line 4).

**Author response image 1. sa2fig1:** Analysis of developing mouse brain tissue. (**A**) Confocal scans of ventricular zones in E12. 5 Mouse developing cortex stained with anti-EGFP, anti-G3BP1, anti-SOX1 and anti-MAP2 antibodies and DAPI. Zoomed-in images of the boxed areas are shown below. Arrows mark G3BP1 positive punctate structures. Scale bar = 50 µm. (**B**) Expression scores of 5’TOP genes *RPL5* and *RPL11* during mouse cortex development. scRNA-seq data from Telley et al. , Science 2019*: http://genebrowser.unige.ch/telagirdon/*.

3) Modify claims or add additional data to address Reviewers' comments. There are several instances in which the authors make statements that are not supported by the data or neglect previously published data, as detailed in the reviews below.

We thank the reviewers for their comment. We have made necessary text changes as indicated below.

a) Additional discussion or experiments should convey the impact of the results on progenitor subtypes and highlight the fact that oRGs are a population that is uniquely subject to mTOR activation.

We thank the reviewers for their comment. We have elaborated this point in the discussion (Page 23 line 28 to Page 24 line 7).

b) Many RBPs have an important role in the regulation of mRNAs during brain development and neuronal differentiation (and are implicated in developmental disorders), including several of the proteins/families listed in Figure 3C. Please provide some examples and references to identify specific examples of proteins in the list that may play a role.

We thank the reviewers for this suggestion. We have added this information in our text (Page 11 line 23-25; Page 12 line 2-10).

c) The results themselves do not suggest that the length of neuron-enriched genes contributes to their complex regulatory mechanisms, but add to the existing literature that should be citedhttps://www.nature.com/articles/nature12504https://academic.oup.com/bioinformatics/article/34/13/i422/5045809https://pubmed.ncbi.nlm.nih.gov/25232122/https://www.nature.com/articles/nature14319https://www.ncbi.nlm.nih.gov/pmc/articles/PMC3638137/

We thank the reviewers for pointing this out and highlighting the studies. We have adapted our text and cited the mentioned studies (Page 13, line 3-8).

d) There are some limitations of the specific reporter and knockout hPSC lines that could be discussed more, as well as providing additional quality control measures including whether the lines remain karyotypically normal throughout the targeting procedure, whether gene expression faithfully recapitulates the activity of the promoters controlling their expression. Specifically, it appears that a significant GFP signal is detected within the neuronal layer (Figure 1B) and that there is a much larger double reporter-positive population than expected (Figure S2A).

We thank the reviewers for their comment. In the revised version of the manuscript, we have added additional data to clarify these points. We have also added more analysis of the dual reporter line, distribution of the GFP and dTomato markers (Figure 1—figure supplement 2) (Page 5 line 11-18) as well as an analysis of the double positive population (Figure 1—figure supplement 5) (Page 7 line 7-17). We have also added karyotyping information on the reporter lines and hPSCs used in this study (new Supplementary File 2).

Reviewer #1 (Recommendations for the authors):This was a nice paper, and generally quite strong. A few questions/suggestions:

We thank the reviewer for the positive and constructive review and comments on our manuscript. We have addressed many of the questions and suggestions as pointed out below.

1. Profiling the "in-between" population would be a very interesting and important characterization with regards to the model of mTOR regulation and the progenitor/neuron transition.

We thank the reviewers for their comment. We have added more analysis on the double positive population (Figure 1—figure supplement 5) (Page 7 line 7-17).

2. The normalization is important, but did it impact the results? Can you validate protein levels in the cases where RNA and protein are dysregulated (ie RNA higher but protein lower and vis versa), specifically by looking more expansively at protein levels?

We agree that normalizations can have an impact on results and their interpretation. Therefore, we show in Figure 1 and Figure 1—figure supplement 5 the absolute TPM and intensity values of the RNAseq and mass spectrometry results, respectively. Furthermore, we independently validated RNA and protein levels by RNA FISH and IHC for the genes RPL5 and RPL11. We agree that additional stainings would be necessary before designing any follow-up studies that would aim to study the discrepancy between RNA and protein levels for specific genes.

3. Subtypes are critical to cortical development. This approach clearly highlights progenitor/neuronal differences but could the observations be extended to one or two subtypes? Specifically, oRGs come to mind as a population that is uniquely subject to mTOR activation.

The reviewer has rightly pointed out that our current strategy highlights progenitor/neuron differences. To focus on subtypes of progenitors and neurons, one would need to opt for single cell analysis or sort specific subpopulations and then perform RNA-protein multiomics. This is currently challenging and would be an important step in the future. Nevertheless, using our temporal data, we can already observe some differences in early stages devoid of outer radial glia and later stages featuring outer radial glia. For instance, 5’TOP transcript-protein discrepancy observed in our data is prominent at early stage and reduces overtime. This is probably linked to the emergence of oRGs with higher mTOR activity and thus not featuring the same regulation as at early stages. This is also reflected in the reduced RNA-stability of 5’TOP transcripts in late-stage progenitors. We have elaborated on this point in the discussion (Page 23 line 28 to Page 24 line 7).

4. The TSC experiments are nice, but also are specific to that pathway and manner of regulation of mTOR signaling. It is possible that mTOR broadly has more complex or nuanced impacts upon 5'-TOP regulation. Thus, I would suggest pharmacologically overactivating and repressing it to explore similar impacts, or limiting the degree to which mTOR is described to overall control the regulation of these transcripts.

We thank the reviewers for their comment and suggestion. Indeed, the mTOR pathway has a complex regulation. Here we used TSC2 KO only as a tool to overactivate mTOR pathway (page 19 line 18-21). However, the native regulation of the mTOR pathway in the tissue remains elusive. The link between mTOR and 5’TOP regulation was based on the previous literature in other cellular systems. Future investigations would shed light on the upstream regulation of mTOR and its downstream effects to gain a molecular understanding of the pathway during corticogenesis. We have mentioned this in the discussion (Page 24 line 13-14).

Two very recent studies from the Giuseppe Testa group have eluded to the role of ribosomal 5’TOP transcript regulation in the pathophysiology of 7q11.23 copy variation disorders and that the perturbation of this regulation seems to also impact the timing of neurogenesis (Mihailovich Biorxiv, 2022; DOI: 10.1101/2022.10.10.511483; Lopez-Tobon Biorxiv 2022; DOI: 10.1101/2022.10.10.511434). These conditions also show a deregulation of the mTOR pathway. Thus, the importance of posttranscriptional regulation of the ribosomal proteins in human brain development and its link with the mTOR pathway is becoming slowly clear. We have also added this information in the discussion of the revised manuscript (Page 23, line 9-13).

Reviewer #3 (Recommendations for the authors):1. The authors identified a stress-related role for processes in corticogenesis, although, without comparison to human tissue, it's possible that some of the results are due to the artificial nature of the organoids as they have been reported to have elevated stress (Bhaduri et al.,). The authors should tone down the statement "Our observation of SG-like RNA-granules in early ventricular radial glia further supports the occurrence of stress-associated processes during corticogenesis," in the discussion as the organoid system does not completely mimic corticogenesis.

We appreciate the reviewer’s concern. We have clarified the ‘stress status’ in the organoids. See above. Additionally, we have amended the text.

2. The data is from organoids from one human stem cell line, the female H9 human embryonic stem cell line and so it is critical to validate the results on 1-2 additional stem cell lines, to rule out the possibility that these results are unique to this one cell line/individual.

See points clarified above.

3. There are several instances in which the authors make statements that are not supported by the data or neglect previously published data.a) Title:a. The use of the word "reveals" in the title implies that post-transcriptional regulation of ribosomal genes has not been seen before, which isn't true, since it is established in other cell types. I think instead the title should reflect that the authors have shown posttranscriptional regulation of ribosomal genes in human neural tissuesi. https://academic.oup.com/nar/article/25/5/995/2360248ii. https://pubmed.ncbi.nlm.nih.gov/11029573/iii. https://pubmed.ncbi.nlm.nih.gov/18498749/b. Since the only validation and focus has been on posttranscriptional regulation of ribosomal genes via 5'TOP, the title also seems too broad unless you add validation demonstrating additional mechanisms of transcriptional regulation of these genes

We have amended the title to better reflect the essence of the study. We believe that the RNA-protein multiomics resource is a major part of the study and has created a useful dataset for the community and necessitates the broad title. We hope that the new title also clarifies that have we identified the posttranscriptional regulation of ribosomal genes in context of human brain organoids.

b) Page 11, Lines 5-8, and Page 11 Lines 13-15"Effective" is not the right word to use here. How can a translation be less effective or more effective? There are more than just the two stated reasons why there could be differences in mRNA transcript level but not protein level in these two modules, some of which are discussed on the next page (page 12, lines 6-10).

We thank the reviewer for pointing this out. We have changed the phrase “effective translation” to “efficient translation”, which is more commonly used. (Page 10, lines 3-4,9,15).

c) Page 12, Lines 22-24: "the RBP motifs we identified and their associated proteins might play a prominent role in cell class-specific expression to promote progenitor or neuron-specific gene regulation " Many RBPs have an important role in the regulation of mRNAs during brain development and neuronal differentiation (and are implicated in developmental disorders), including several of the proteins/families listed in Figure 3C. It might be more impactful to specifically provide some examples and references instead of generically stating that the proteins in the list "might" play a role. These references would also strengthen your claims and provide additional evidence that your data and model system are consistent with previously published work in the field.

We appreciate the comment and have added this information to our text. (Page 11 line 23-25; Page 12 line 2-10).

d) Given that alternative splicing is mentioned in the introduction (page 5, line 9) as something that has a role in determining progenitor fate and neuronal migration in mouse and given that the RBPs in Figure 3C contains several splicing factors/regulators (SRSF family, KHDRBS3, CPEB3, PTBP1, etc.), it might be worth discussing this more or even adding splicing analysis into your paper. It could provide additional clarity about the mRNA and protein levels in the applicable modules.

We appreciate the comment. However, we feel splicing analysis is currently out of the scope of this paper, but agree that it is indeed a very interesting approach to follow in a future study. Hence, we have explicitly mentioned this in the newly added ‘limitations of the study’ section (page 25 line 1922).

e) Page 13, Line 1-3Omit "might" from this sentence because, in the next sentence, you give a very definitive example of a fate regulator that is regulated by miRNAs. You could alternatively say that these data are consistent with miRNA regulation of their expression, for example.

We have amended the text. (Page 12 line 14)

f) Page 13, Line 20-22These observations themselves do not suggest that the length of neuron-enriched genes contributes to their complex regulatory mechanisms. These observations add to the existing literature that previously suggested this:https://www.nature.com/articles/nature12504https://academic.oup.com/bioinformatics/article/34/13/i422/5045809https://pubmed.ncbi.nlm.nih.gov/25232122/https://www.nature.com/articles/nature14319https://www.ncbi.nlm.nih.gov/pmc/articles/PMC3638137/

We have amended the text to incorporate these references. (Page 13, 3-8)

g) The conclusions about the results of Figure 3 (and its supplements) could likely be strengthened by performing similar analyses on the datasets used for Figure 4 Supplemental Figure 1 (Blair et al. 2017; Eze et al. 2021)

The reviewer has made a very interesting suggestion. However, we feel this would require a separate study on its own, to identify cell type specific gene expression modules present in 2D culture (Blair et al. 2017) or the fetal scRNA-seq dataset (Eze et al., 2021). These analyses would most likely not result in the same modules that have been analyzed here, which would make a comparative analysis challenging. We therefore feel that such an analysis would be outside the scope of this study.