# Supplementary figures and images for: Integrated transcriptome and proteome analysis reveals posttranscriptional regulation of ribosomal genes in human brain organoids
Source: eLife. 2023 Mar 29;12:e85135. doi: 10.7554/eLife.85135 (PMC10059687; doi:10.7554/eLife.85135)

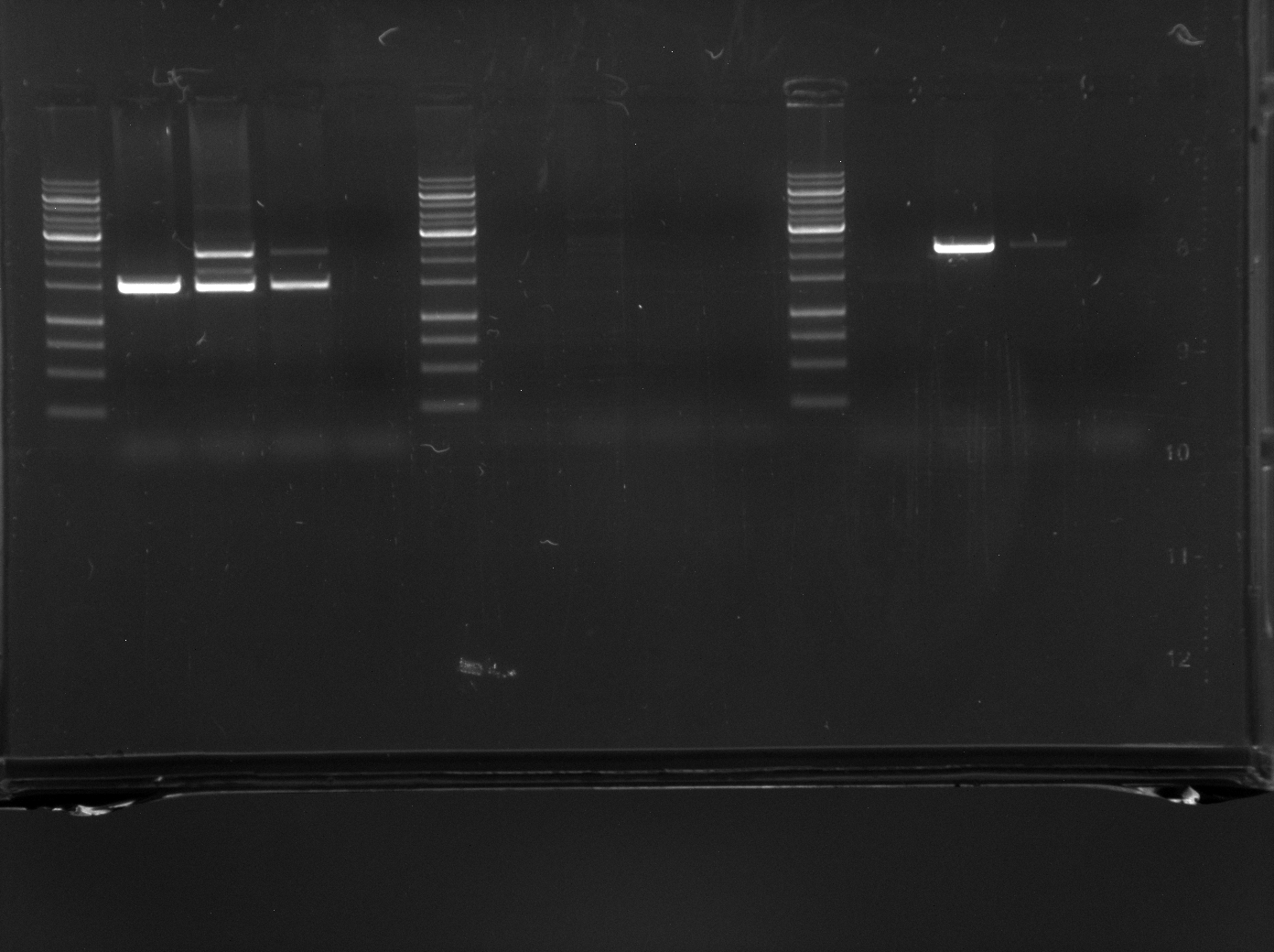

Supplement: Figure 1—figure supplement 1—source data 1. [file elife-85135-fig1-figsupp1-data1.zip › Figure 1-figure supplement 1-source data 1.tif]

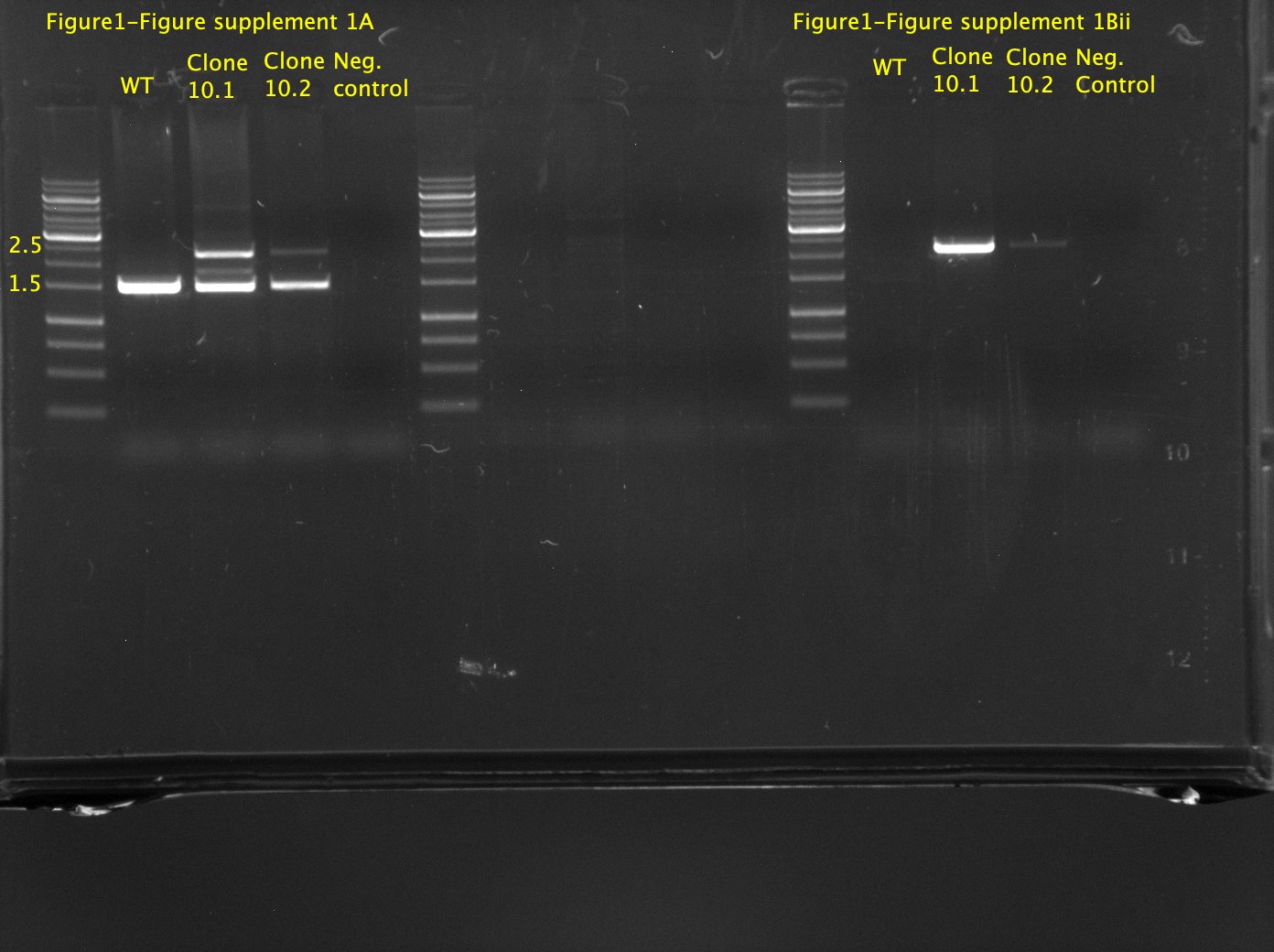

Supplement: Figure 1—figure supplement 1—source data 2. [file elife-85135-fig1-figsupp1-data2.zip › Figure 1-figure supplement 1-source data 2.jpg]

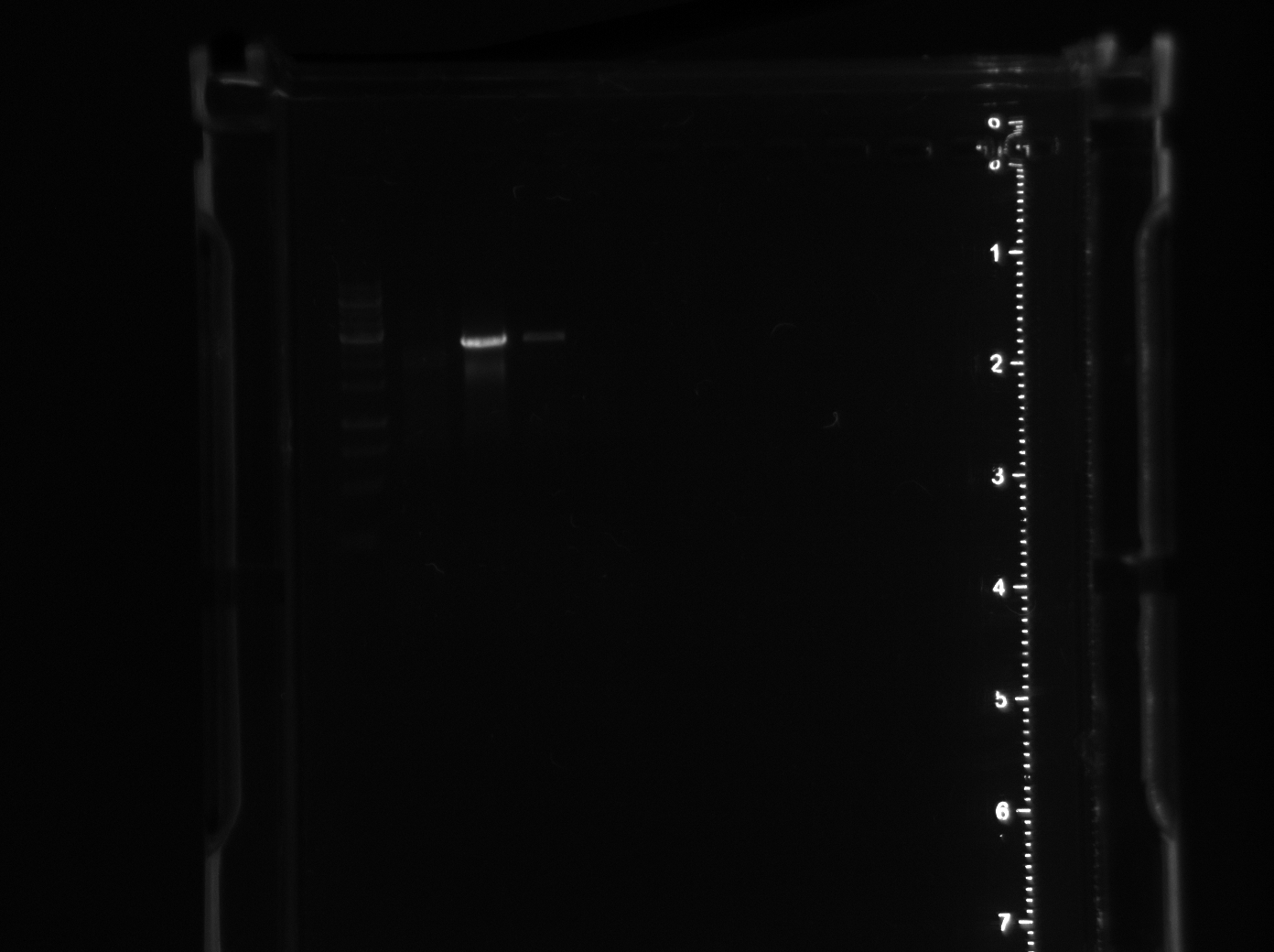

Supplement: Figure 1—figure supplement 1—source data 3. [file elife-85135-fig1-figsupp1-data3.zip › Figure 1-figure supplement 1-source data 3.tif]

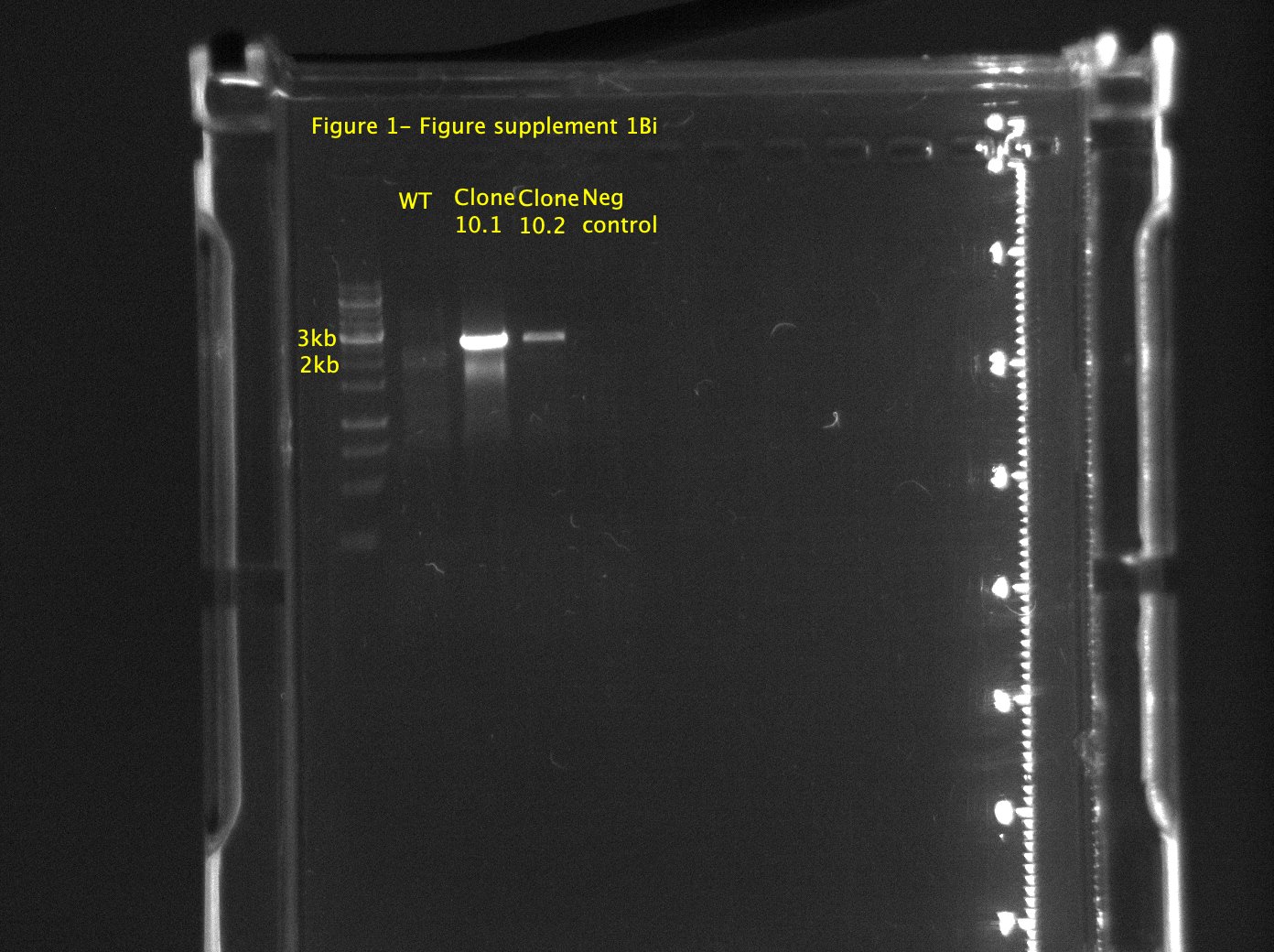

Supplement: Figure 1—figure supplement 1—source data 4. [file elife-85135-fig1-figsupp1-data4.zip › Figure 1-figure supplement 1-source data 4.jpg]

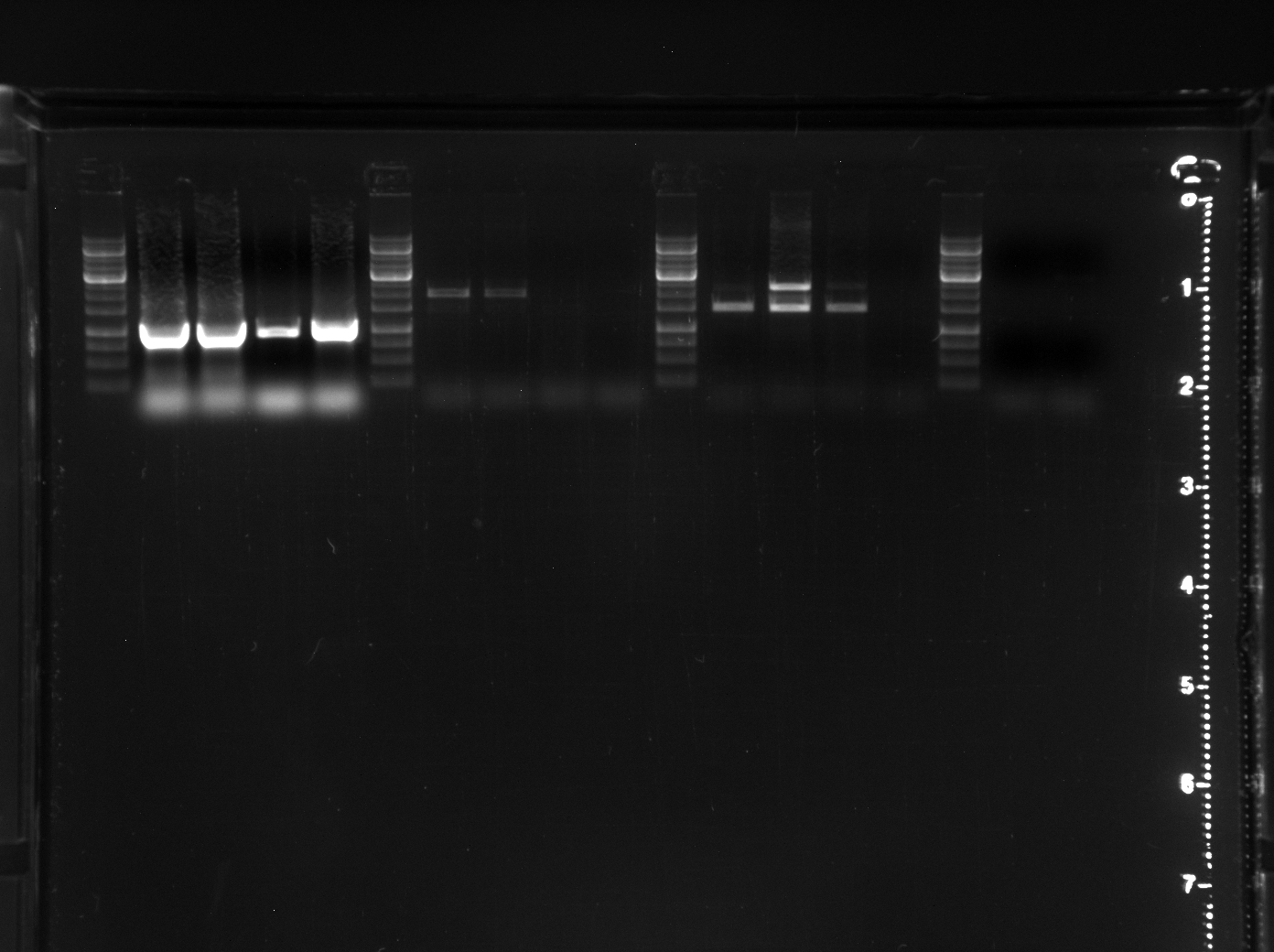

Supplement: Figure 1—figure supplement 1—source data 5. [file elife-85135-fig1-figsupp1-data5.zip › Figure 1-figure supplement 1-source data 5.tif]

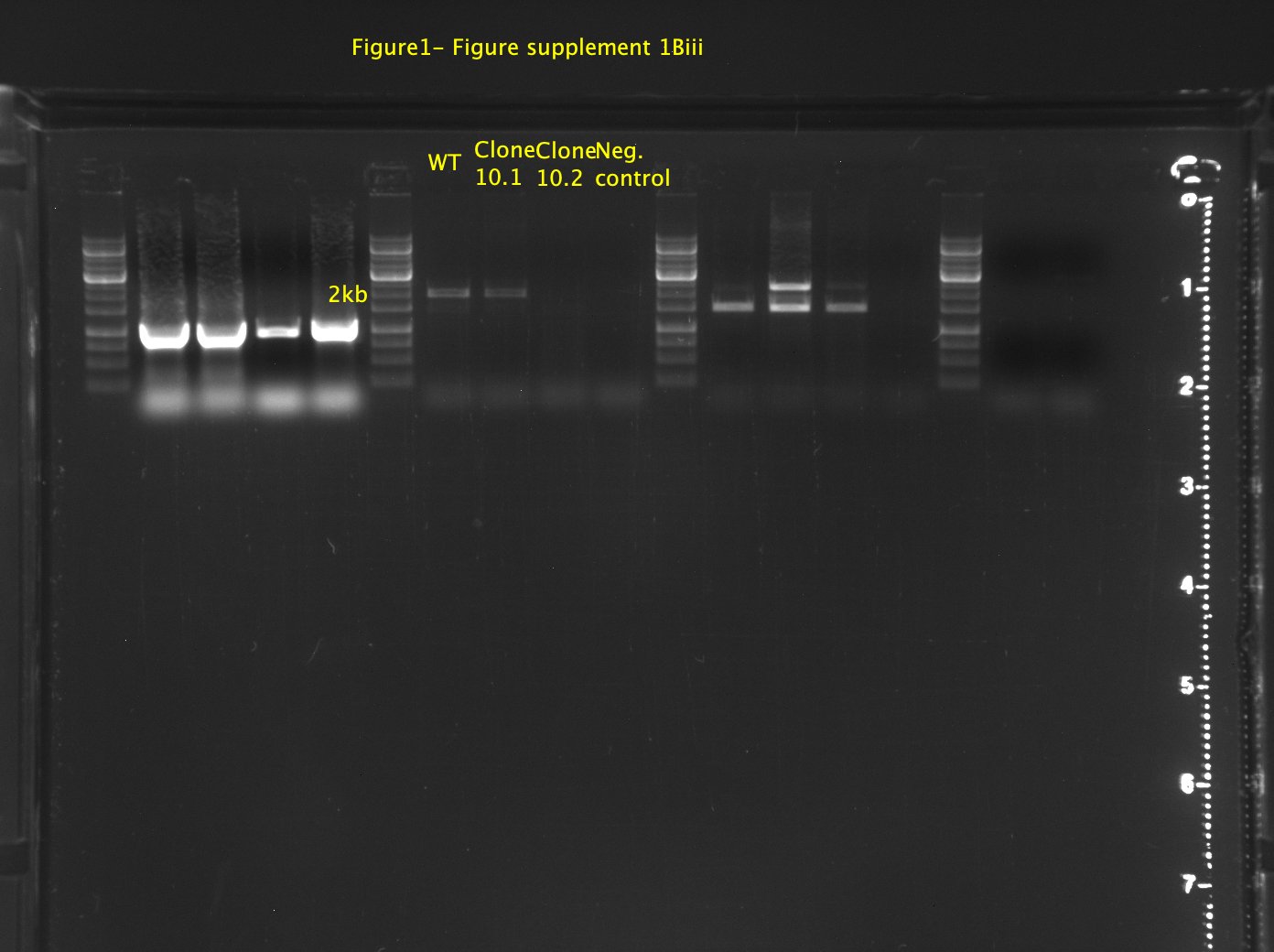

Supplement: Figure 1—figure supplement 1—source data 6. [file elife-85135-fig1-figsupp1-data6.zip › Figure 1-figure supplement 1-source data 6.jpg]

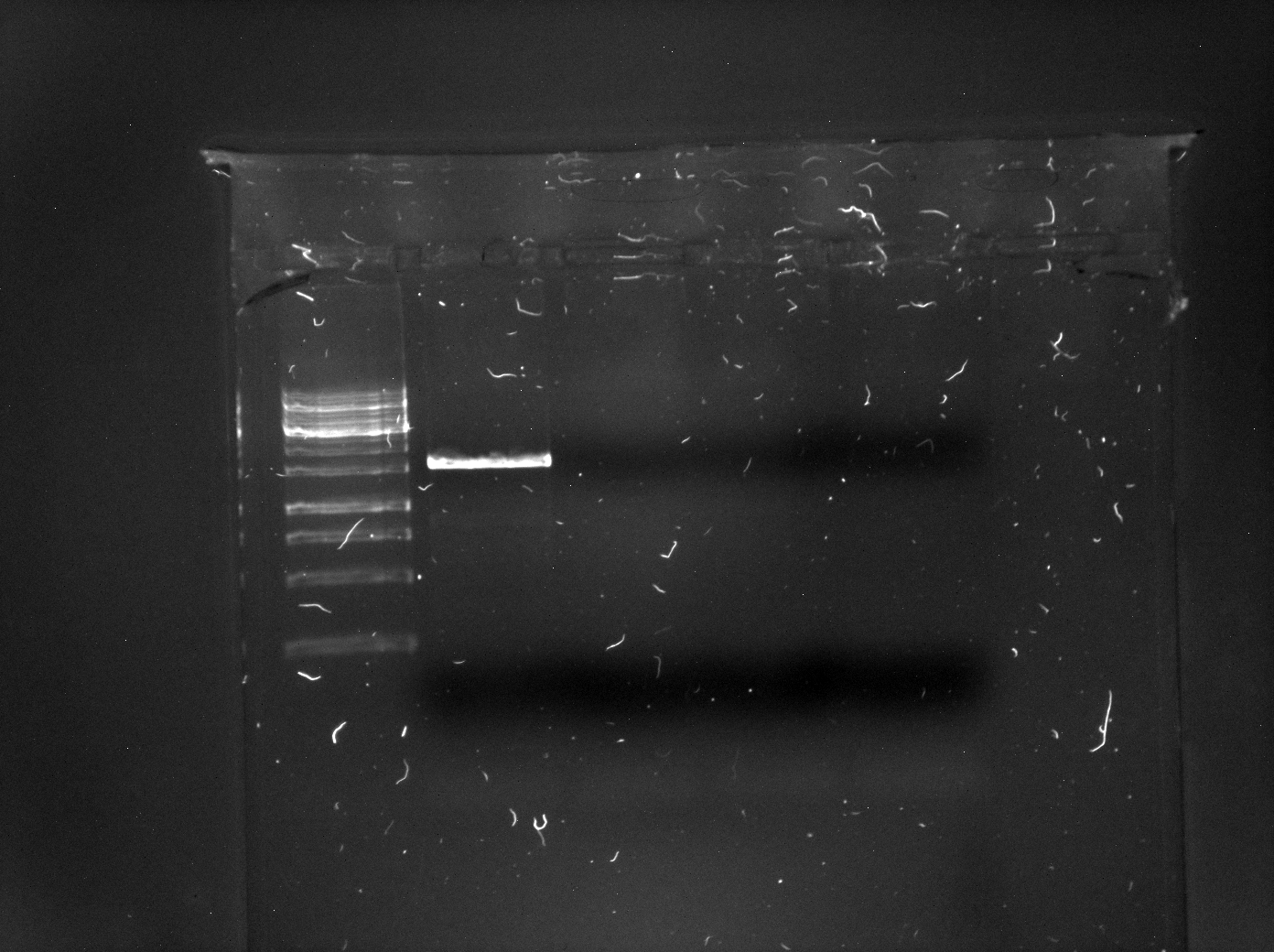

Supplement: Figure 4—figure supplement 5—source data 1. [file elife-85135-fig4-figsupp5-data1.zip › Figure 4-figure supplement 5-source data 1.tif]

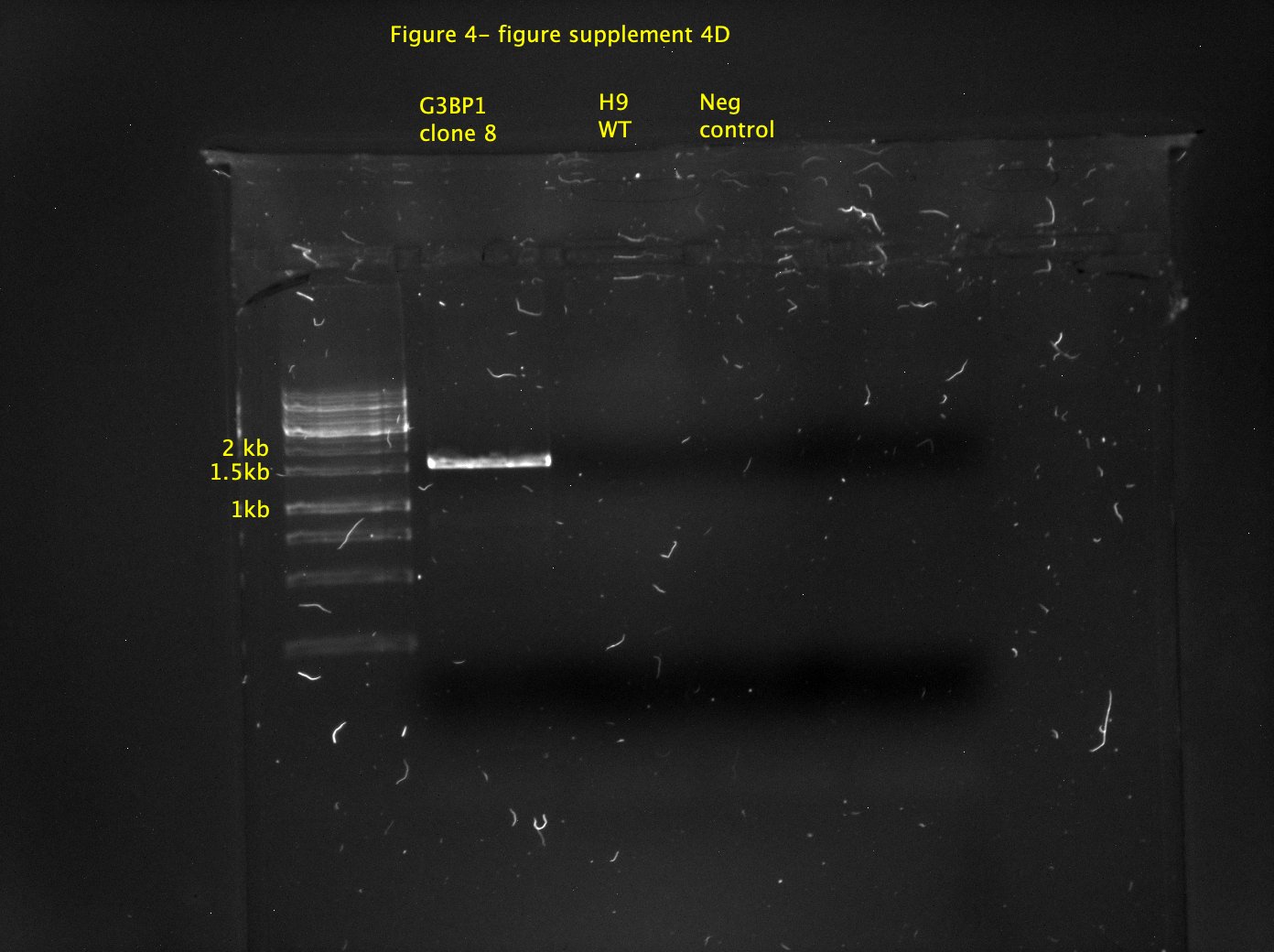

Supplement: Figure 4—figure supplement 5—source data 1. [file elife-85135-fig4-figsupp5-data1.zip › Figure 4-figure supplement 5-source data 2.jpg]
