# Supplementary material for: Integrated transcriptome and proteome analysis reveals posttranscriptional regulation of ribosomal genes in human brain organoids
Source: eLife. 2023 Mar 29;12:e85135. doi: 10.7554/eLife.85135 (PMC10059687; doi:10.7554/eLife.85135)
Supplement: Supplementary file 2. [file elife-85135-supp2.pdf]

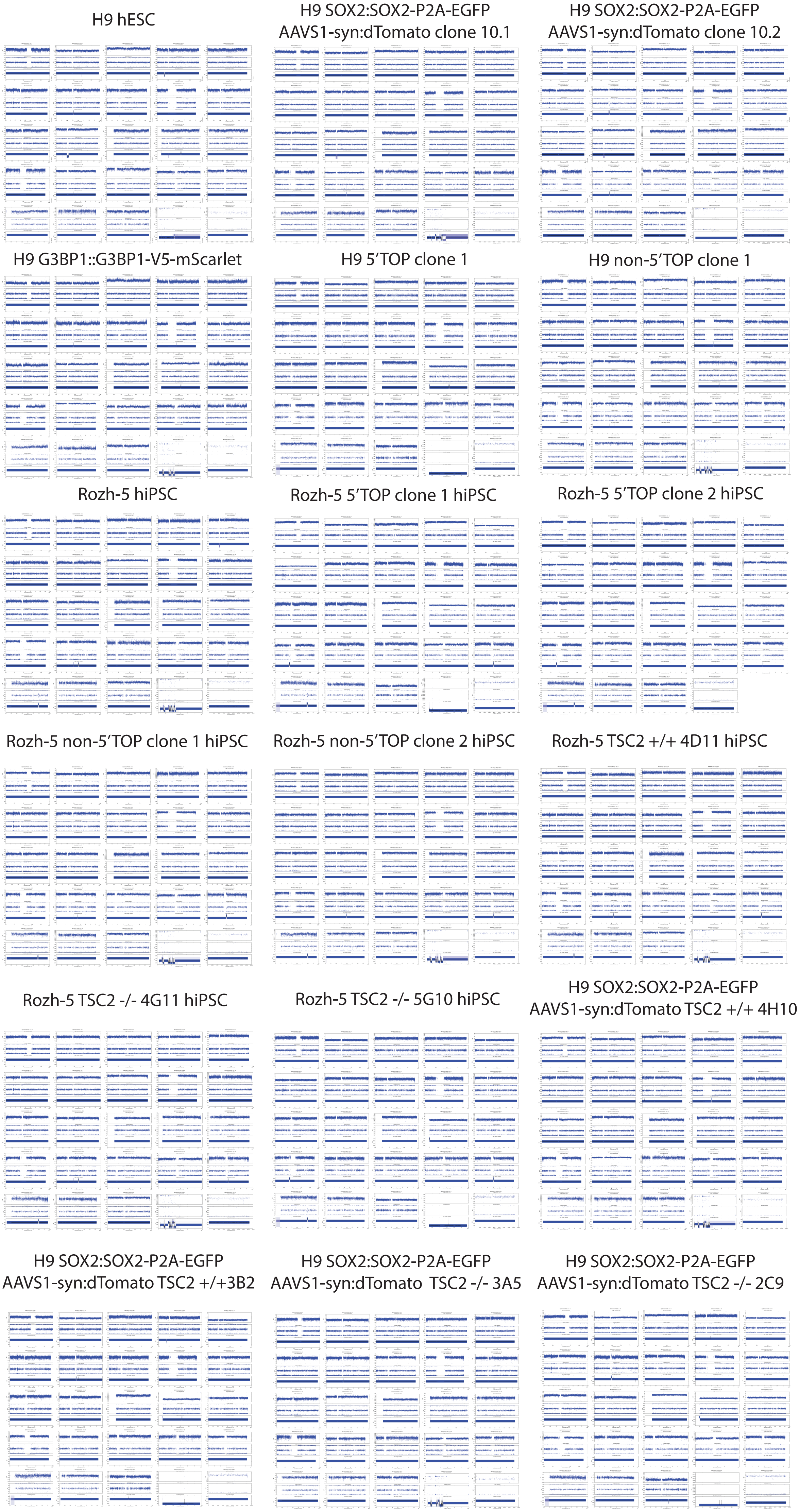
H9 hESC
H9 SOX2:SOX2-P2A-EGFP AAVS1-syn:dTomato clone 10.1
H9 SOX2:SOX2-P2A-EGFP AAVS1-syn:dTomato clone 10.2
H9 G3BP1::G3BP1-V5-mScarlet
H9 5'TOP clone 1
H9 non-5'TOP clone 1
Rozh-5 hiPSC
Rozh-5 5'TOP clone 1 hiPSC
Rozh-5 5'TOP clone 2 hiPSC
Rozh-5 non-5'TOP clone 1 hiPSC
Rozh-5 non-5'TOP clone 2 hiPSC
Rozh-5 TSC2 +/+ 4D11 hiPSC
Rozh-5 TSC2 -/- 4G11 hiPSC
Rozh-5 TSC2 -/- 5G10 hiPSC
H9 SOX2:SOX2-P2A-EGFP AAVS1-syn:dTomato TSC2 +/+ 4H10
H9 SOX2:SOX2-P2A-EGFP AAVS1-syn:dTomato TSC2 +/+3B2
H9 SOX2:SOX2-P2A-EGFP AAVS1-syn:dTomato TSC2 -/- 3A5
H9 SOX2:SOX2-P2A-EGFP AAVS1-syn:dTomato TSC2 -/- 2C9
